# PKD1 Mediates Negative Feedback of PI3K/Akt Activation in Response to G Protein-Coupled Receptors

**DOI:** 10.1371/journal.pone.0073149

**Published:** 2013-09-09

**Authors:** Yang Ni, James Sinnett-Smith, Steven H. Young, Enrique Rozengurt

**Affiliations:** 1 Division of Digestive Diseases, Department of Medicine, David Geffen School of Medicine, CURE: Digestive Diseases Research Center and Molecular Biology Institute, University of California at Los Angeles, Los Angeles, California, United States of America; 2 Department of Thoracic Surgery, Provincial Hospital Affiliated to Shandong University, Jinan, China; Lerner Research Institute, United States of America

## Abstract

We examined whether protein kinase D1 (PKD1) mediates negative feeback of PI3K/Akt signaling in intestinal epithelial cells stimulated with G protein-coupled receptor (GPCR) agonists. Exposure of intestinal epithelial IEC-18 cells to increasing concentrations of the PKD family inhibitor kb NB 142­70, at concentrations that inhibited PKD1 activation, strikingly potentiated Akt phosphorylation at Thr^308^ and Ser^473^ in response to the mitogenic GPCR agonist angiotensin II (ANG II). Enhancement of Akt activation by kb NB 142-70 was also evident in cells with other GPCR agonists, including vasopressin and lysophosphatidic acid. Cell treatment rovincial Hospital Affiliated to Shandong University, Jinan, China with the structurally unrelated PKD family inhibitor CRT0066101 increased Akt phosphorylation as potently as kb NB 142–70. Knockdown of PKD1 with two different siRNAs strikingly enhanced Akt phosphorylation in response to ANG II stimulation in IEC-18 cells. To determine whether treatment with kb NB 142–70 enhances accumulation of phosphatidylinositol (3,4,5)-trisphosphate (PIP_3_) in the plasma membrane, we monitored the redistribution of Akt-pleckstrin homology domain-green fluorescent protein (Akt-PH-GFP) in single IEC-18 cells. Exposure to kb NB 142–70 strikingly increased membrane accumulation of Akt-PH-GFP in response to ANG II. The translocation of the PIP_3_ sensor to the plasma membrane and the phosphorylation of Akt was completed prevented by prior exposure to the class I p110α specific inhibitor A66. ANG II markedly increased the phosphorylation of p85α detected by a PKD motif-specific antibody and enhanced the association of p85α with PTEN. Transgenic mice overexpressing PKD1 showed a reduced phosphorylation of Akt at Ser^473^ in intestinal epithelial cells compared to wild type littermates. Collectively these results indicate that PKD1 activation mediates feedback inhibition of PI3K/Akt signaling in intestinal epithelial cells *in vitro* and *in vivo*.

## Introduction

The phosphoinositide 3-kinase (PI3K)/Akt pathway plays a critical role in regulating a plehora of biological processes, including metabolism, migration, survival, autophagy and growth [1], [2]. In response to growth factor-activated tyrosine kinase receptors, PI3K catalyzes the formation of phosphatidylinositol (3,4,5)-trisphosphate (PIP_3_), a membrane lipid second messenger that coordinates the localization and activation of a variety of downstream effectors the most prominent of which are the isoforms of the Akt family [3]. The Akts possess a PH domain and conserved residues (Thr^308^ and Ser^473^ in Akt1, the most commonly expressed isoform in normal cells) which are critical for Akt activation. Specifically, Akt translocated to the plasma membrane in response to products of PI3K, is activated by phosphorylation at Thr^308^ by PDK1 and at Ser^473^ by mTORC2 [4], [5].

In contrast to the canonical pathway activated by tyrosine kinase receptors, the effect of G protein-coupled receptor (GPCR) activation on PI3K/Akt signaling is less well defined and appears to depend on cell context. In some epithelial cell systems, GPCR agonists, including angiotensin II, muscarinic agonists and PGE_2_, induce rapid but transient Akt activation, at least in part, via EGFR transactivation [6]–[9]. Intriguingly, activation of certain Gq-coupled receptors and Gq proteins has also been shown to inhibit Akt in a variety of cell types [10]–[15]. Although activation of Akt is required for fundamental processes, constitutive activation of Akt promotes senescence in a variety of cell types [16], mitochondrial disfunction [17], [18] and growth arrest [19]. Reciprocally, PI3K/Akt inhibition induces the expression and activation of multiple tyrosine kinase receptors [20], [21]. Therefore, negative feedback regulation of Akt activity by activated GPCRs may play an important role in cell regulation but the mechanism(s) involved remain largely unknown.

Protein kinase D1 (PKD1), the founding member of a new protein kinase family within the CAMK group [22], has emerged as prominent downstream signal induced by activated GPCRs that act through G_q_, G_12_, G_i_, and Rho [22]–[30]. PKD1 is rapidly activated through protein kinase C (PKC)-mediated phosphorylation of Ser^744^ and Ser^748^ in the PKD1 activation loop [31]–[33]. PKD1 catalytic activation within cells leads to its autophosphorylation at Ser^916^ [34]–[37]. Rapid PKC-dependent PKD1 activation is followed by a late, PKC-independent phase of activation induced by G_q_-coupled receptor agonists [36]–[38]. Accumulating evidence demonstrate that the PKD family plays an important role in several cellular processes and activities [23], including stimulation of DNA synthesis and proliferation [35]–[37], [39]–[41]. Indeed, PKD1 activation plays a critical role in mediating GPCR-induced migration and proliferation in non-transformed intestinal epithelial IEC-6 and IEC-18 cells [37], [42]. In these cells, we also demonstrated rapid and transient Akt activation via GPCR-induced EGFR transactivation [6]. Recent studies demonstrated that cell treatment with the phorbol ester PMA inhibited PI3K activation via phosphorylation of the p85α regulatory subunit by PKD1 [43]. However, these studies used ectopically expressed proteins and phorbol esters, which are ultrapotent, non-physiological surrogates of rapidly turning over endogenous DAG. Subsequently, it was reported that PKD1 enhances interaction between p85α and PTEN (PI3K-phosphatase and tensin homolog deleted on chromosome 10) in neutrophils [44]. However, the impact of PKD1 activity on the cellular levels of PIP_3_ has not been examined in any cell type.

In the present study, we used non-transformed intestinal epithelial cells stimulated via GPCRs as a model system to examine whether PKD1 negatively regulates PI3K/Akt signaling and cellular levels of PIP_3_. Our results demonstrate that PKD1 mediates potent feedback inhibition of PIP_3_/Akt activation in intestinal epithelial IEC-18 cells stimulated with multiple GPCR agonists.

## Materials and Methods

### Ethics Statement

This study was carried out in strict accordance with the recommendations in the Guide for the Care and Use of Laboratory Animals of the National Institutes of Health. The protocol was approved by the Animal Research Committee of the University of California, Los Angeles (Protocol Number: 2001-142–23).

### Akt phosphorylation in intestinal epithelial cells in vivo

To assess the effect of PKD1 on Akt phosphorylation *in vivo*, we used transgenic mice that express elevated PKD1 protein in the small intestine and colonic epithelium and control littermates. The generation of PKD1 transgenic mice was described elsewhere [37]. To perform anatomical dissection and tissue collection mice were first euthanized in a CO_2_ chamber. Overexpression of PKD1 in the ileum was verified using epithelial cells isolated sequentially along the crypt-villus axis by timed incubations in EDTA-PBS solutions. To measure PKD1 expression and Akt phosphorylation, lysates of intestinal cells isolated from gender- and age-matched mice (4 PKD1 transgenic mice and 4 nontransgenic littermates) were subjected to immunoblotting, as described above.

### Cell Culture

The non-transformed rat intestinal epithelial IEC-6 and IEC-18 cells [45], [46], originated from intestinal crypt cells, have provided a model system to examine migration, proliferation and differentiation [42]. Intestinal epithelial IEC-18 cells were purchased from ATCC and maintained as described in [36], [37]. Briefly, cells were cultured in Dulbecco's modified Eagle's medium (DMEM) supplemented with 10% fetal bovine serum (FBS) and penicillin-streptomycin, and kept at 37°C in a 10% CO_2_ incubator. Stock cultures were sub-cultured every 3–4 days.

### Immunoblotting and detection of Akt, S6K and PKD1 phosphorylation

Serum-starved, confluent intestinal epithelial IEC-18 cells treated with inhibitors and/or agonists were lysed in 2x SDS-polyacrylamide gel electrophoresis (PAGE) sample buffer (20 mM Tris/HCl, pH 6.8, 6% SDS, 2 mM EDTA, 4% 2-mercaptoethanol, 10% glycerol) and boiled for 10 min. After SDS-PAGE, proteins were transferred to Immobilon-P membranes. The transfer was carried out at 100 V, 0.4 A at 4°C for 4 h using a Bio-Rad transfer apparatus. The transfer buffer consisted of 200 mM glycine, 5 mM Tris, 0.01% SDS, and 20% CH_3_OH. For detection of proteins, membranes were blocked using 5% nonfat dried milk in PBS (pH 7.2) and then incubated for at least 2 h with the desired antibodies diluted in PBS containing 3% nonfat dried milk. Primary antibodies bound to immunoreactive bands were visualized by enhanced chemiluminescence (ECL) detection with horseradish peroxidase-conjugated anti-mouse, anti-rabbit antibody and a FUJI LAS-4000 Mini Luminescent Image Analyzer. The phosphospecific polyclonal antibodies used (pSer^473^, pThr^308^, pThr^389^, pS^916^) detect the phosphorylated state of residues Ser^473^ and Thr^308^ of Akt, Thr^389^ of S6K and Ser^916^ of PKD.

### Knockdown of PKD1 levels via siRNA transfection

The Stealth siRNA duplexes were purchased from Invitrogen (Carlsbad, CA) Two different PKD1 siRNAs were designed to target the mRNA of rat PKD1 (XM_234108): Oligo 1 GAACCUUCAUCACCCUGGUUGUA; Oligo2, GAGAAGAGGUCAAAUUCGCAGUCAU. For siRNA transfection the reverse transfection method was used, the siRNA pool was mixed with Lipofectamine RNAiMAX (Invitrogen, Carlsbad, CA) according to the manufacturer's protocol and added to 35mm dishes. IEC-18 cells were then plated on top of the siRNA/Lipofectamine RNAiMAX complex at a density of 1×10^5^ cells/35 mm dish. Control transfections were carried out with Stealth siRNA neagative control (Invitrogen, Carlsbad, CA). Four days after transfection, cells were used for experiments and subsequent Western blot analysis.

### Cell transfection

IEC-18 cells were transfected with the plasmid containing a cDNA encoding a green fluorescent protein (GFP) tagged-Akt pleckstrin homology domain (Akt-PH-GFP) from Addgene (pcDNA3-Akt-PH-GFP cat #18836) by using Lipofectamine Plus (Invitrogen) as suggested by the manufacturer. Analysis of the cells transiently transfected were performed 24 h after transfection.

### Real-time GFP-AKT-PH imaging in single live cells

Single live-cell imaging of the GFP tagged Akt-PH domain was achieved with a fluorescence microscope. The microscope used was a Zeiss (Carl Zeiss, Inc.) epifluorescent Axioskop with a Zeiss Achroplan 40×/0.75 water immersion objective (Zeiss). Images were captured as uncompressed 24-bit TIF files with a Pusuit cooled single CCD color digital camera driven by SPOT software (Diagnostic Instruments, Sterling Heights, MI). GFP fluorescence was observed with a HI Q filter set for fluorescein isothiocyanate (Chroma Technology, Rockingham, VT).

Quantitative analysis of the relative change in plasma membrane and cytosol fluorescence intensity of individual cells were performed by importing the TIF images into Zeiss LSM 510 software and performing profile scans with the largest line width. Five equally spaced line profiles were taken for each cell or cell pair. Intensities were background corrected, and the intensities at the membrane were divided by those in the immediately surrounding cytoplasm. We analyzed 20–30 cells in each experiment, and each experiment was performed in duplicate. The selected cells displayed in the figures were representative of 90% of the population of positive cells.

### Immunoprecipitation of the PI3K p85α subunit

Confluent IEC-18 cells were lysed in buffer A containing 50 mM Tris-HCl, pH 7.6, 2 mM EGTA, 2 mM EDTA, 1 mM dithiothreitol, 100 µg/ml leupeptin, 1 mM 4-(2-aminoethyl)-benzenesulfonyl fluoride, hydrochloride (Pefabloc) and 1% Triton X-100. p85α was immunoprecipitated with p85α rabbit monoclonal antibody (Cell Signaling Technology). The immune complexes were recovered using protein-A coupled to agarose and analyzed for PKD1-mediated phosphorylation (using a PKD1 motif antibody) or complex formation with tyrosine phosphorylated EGFR, p110α or PTEN, as described in Fig. 7.

### Akt phosphorylation in intestinal epithelial cells *in vivo*

To assess the effect of PKD1 on Akt phosphorylation *in vivo*, we used transgenic mice that express elevated PKD1 protein in the small intestine and colonic epithelium and control littermates. The generation of PKD1 transgenic mice was described elsewhere [37]. To perform anatomical dissection and tissue collection mice were euthanized in a CO2 chamber. Overexpression of PKD1 in the ileum was verified using epithelial cells isolated sequentially along the crypt-villus axis by timed incubations in EDTA-PBS solutions. To measure PKD1 expression and Akt phosphorylation, lysates of intestinal cells isolated from gender- and age-matched mice (4 PKD1 transgenic mice and 4 nontransgenic littermates) were subjected to immunoblotting, as described above. This study was carried out in strict accordance with the recommendations in the Guide for the Care and Use of Laboratory Animals of the National Institutes of Health. The protocol was approved by the Animal Research Committee of the University of California, Los Angeles (Protocol Number: 2001-142–23).

### Materials

DMEM was obtained from Invitrogen (Carlsbad, CA). Angiotensin II, vasopressin and LPA were obtained from Sigma Chemical (St. Louis, MO). kb NB 142-70 was obtained from R&D Systems (Minneapolis, MN) and CRT0066101 (41) was obtained from Cancer Research Technology Discovery Laboratories (London, UK). Anti-phosphotyrosine 4G10 antibody was purchased from Millipore All other antibodies were purchased from Cell Signaling Technology (Danvers, MA). All other reagents were of the highest grade available.

## Results

### Exposure of IEC-18 cells to the selective PKD family inhibitor kb NB 142-70 potentiates GPCR-induced Akt activation

**Figure 1:**
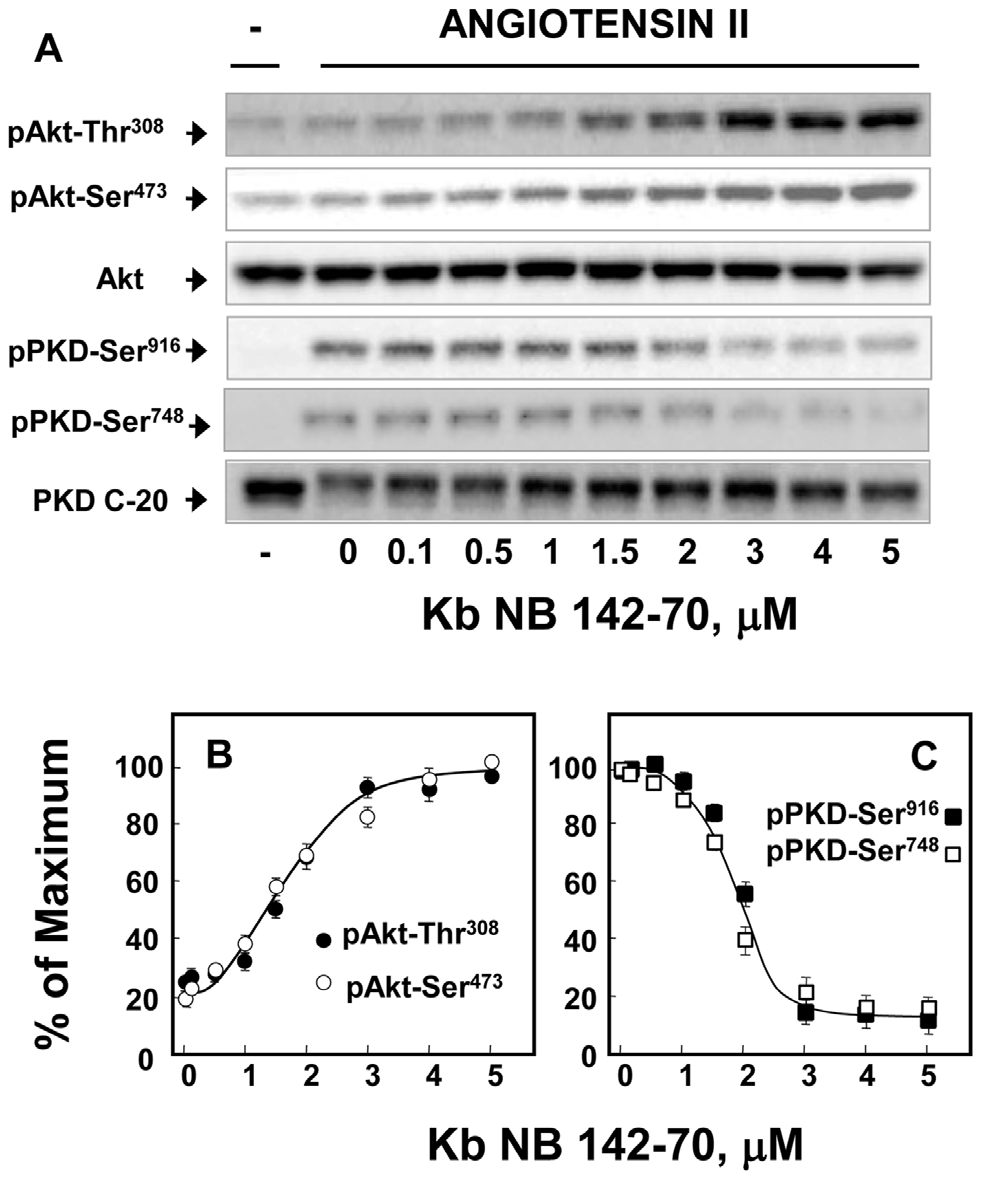
PKD1 inhibition by increasing concentrations of kb NB 142-70 potentiates PI3K/Akt activation in angiotensin II-stimulated IEC-18 cells. A, Confluent cultures of IEC-18 cells were incubated in the in the absence (−) or presence (kb) of increasing concentrations of kb NB 142–70 for 1 h prior to stimulation of the cells without (−) or with 50 nM angiotensin II for 1 h, as indicated. All cultures were then lysed with 2×SDS–PAGE sample buffer. The samples were analyzed by SDS-PAGE and immunoblotting with antibodies that detect the phosphorylated state of Akt at Ser^473^, Akt at Thr^308^ and total Akt to verify equal gel loading and PKD1 at Ser^916^ and Ser^748^ **B and C**, Quantifications were performed by using Multi Gauge V3.0 and plotted as a percentage of the maximum response, mean ± S.E n = 3, induced by angiotensin II and kb NB 142–70 **(B)** or as percentage of the maximum response, mean ± S.E n = 3, induced by angiotensin II **(C)**.

Stimulation of intestinal epithelial IEC-18 cells with angiotensin II (ANG II), a mitogenic agonist that activates Gq-coupled receptors endogenously expressed by these cells, induced a detectable increase in Akt activation, as judged by its phosphorylation at either Ser^473^, the residue phosphorylated by mTORC2 or Thr^308^, the residue in the activation loop phosphorylated by PDK1 (**Fig** **1** **A**). The level of Akt phosphorylation on these residues in response to ANG II was small, as compared with the effect induced by either EGF or insulin in parallel cultures (**Fig.** **S 1**). Because ANG II in contrast to EGF or insulin induces robust PKD1 activation in IEC-18 cells (**Fig.** **S 1**), we examined whether ANG II-induced Akt phosphorylation was restrained by feedback inhibition mediated by PKD1. Cultures of IEC-18 cells were treated with increasing concentrations of the selective PKD family inhibitor kb NB 142–70 for 1 h and then challenged with 50 nM ANG II [42]. As shown in **Fig** **1** **A**, prior exposure of IEC-18 cells to kb NB 142–70 strikingly potentiated Akt phosphorylation at Thr^308^ and Ser^473^ in a dose-dependent manner. Half-maximal potentiation was obtained at a concentration of 1.5 µM (**Fig** **1** **B**). The enhancements of Akt phosphorylation ocurred at concentrations of kb NB 142-70 that inhibited PKD1 activation in the same cells, as scored by PKD1 autophosphorylation at either Ser^916^ in the C-terminus or Ser^748^ in the activation loop (**Fig.** **1** **, A;** quantification in **Fig.** **1** **, C**).

**Figure 2:**
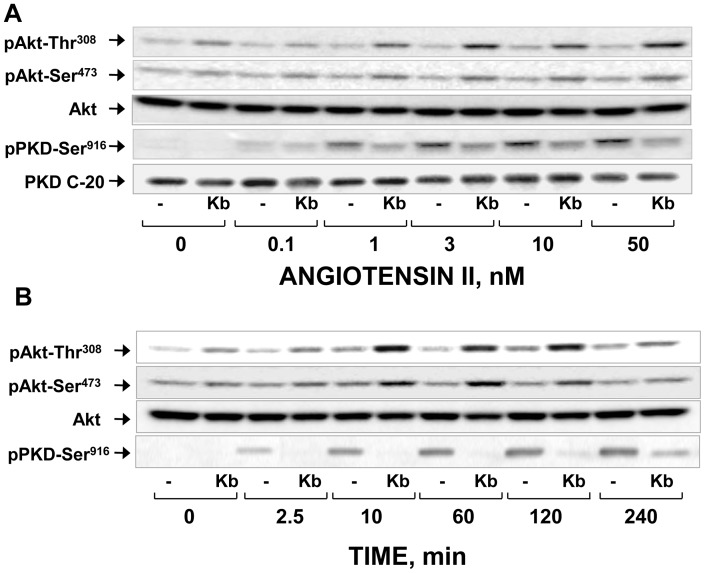
Dose and time dependent potentiation of PI3K/Akt activation by kb NB 142–70 in angiotensin II-stimulated IEC-18 cells. Confluent cultures of IEC-18 cells were incubated in the absence (−) or presence 3.5 µM kb NB 142–70 (kb) for 1 h prior to stimulation of the cells with increasing concentrations of angiotensin II for 1 h (**panel A**) with 50 nM angiotensin II for the indicated times (**panel B**). In all cases, the cultures were lysed with 2× SDS–PAGE sample buffer. The samples were then analyzed by SDS-PAGE and immunoblotting with antibodies that detect the phosphorylated state of Akt at Ser^473^ and Thr^308^, total Akt to verify equal gel loading and PKD1 at Ser^916^.

The striking increase of Akt phosphorylation at Thr^308^ and Ser^473^ by treatment with kb NB 142–70 was also observed in IEC-18 cells stimulated with various concentrations of ANG II (0.1–50 nM); enhancement was detected in cells stimulated with ANG II at a concentration as low as 1 nM (**Fig.** **2** **, A**). ANG II induced PKD1 activation (scored by Ser^916^ autophosphorylation) at similar concentrations. Enhancement of Akt activation by addition of kb NB 142–70 reached a maximum as early as 10 min after ANG II stimulation and persisted at the maximal level for at least 2 h (**Fig.** **2** **, B**). We verified that treatment with kb NB 142–70 suppressed PKD1 autophosphorylation on Ser^916^ at all concentrations of ANG II (**Fig.** **2** **, A**) and times examined (**Fig.** **2** **, B**).

**Figure 3:**
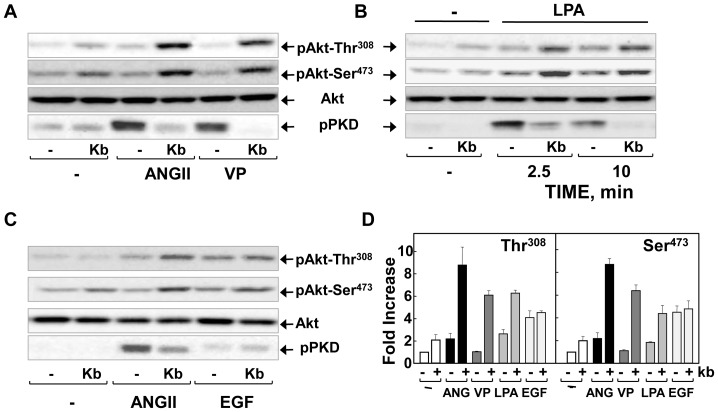
PKD1 inhibition potentiates PI3K/Akt activation in IEC-18 cells stimulated with vasopressin and LPA. Confluent cultures of IEC-18 cells were incubated in the in the absence (−) or presence 3.5 µM kb NB 142–70 (kb) for 1 h prior to stimulation of the cells with either 50 nM angiotensin II (ANGII) or 50 nM vasopressin (VP) for 1 h (**panel A**)**,** 10 µM LPA for 2.5 or 10 min (**panel B**)**,** 50 nM angiotensin II (ANGII) or 50 ng/ml EGF for 1 h (**panel C**)**.** All cultures were then lysed with 2× SDS–PAGE sample buffer. The samples were analyzed by SDS-PAGE and immunoblotting with antibodies that detect the phosphorylated state of Akt at Ser^473^ and Thr^308^, total Akt to verify equal gel loading and PKD1 at Ser^916^ (p-PKD). **Panel D,** Fold increases following PKD1 inhibition for angiotensin II (ANG, n = 20), vasopressin (VP, n = 4), LPA (n = 4) and EGF (n = 3) in Akt at Thr^308^ and Ser^473^ phosphorylation were quantified using Multi Gauge V3.0 and plotted as bars, mean ± S.E.

We next determined whether inhibition of PKD1 enhances Akt phosphorylation in IEC-18 cells stimulated via GPCRs other than those that bind ANG II. The agonists vasopressin and LPA induced robust PKD activation in IEC-18 cells (**Fig** **3**). Exposure to kb NB 142–70 enhanced Akt phosphorylation and inhibited PKD1 activation in IEC-18 cells stimulated with either vasopressin (**Fig** **3**, **A**) or LPA (**Fig** **3**, **B**) as potently as in paralell cultures stimulated with ANG II. In contrast, prior exposure to kb NB 142–70 had only a small potentiating effect on Akt activation induced by EGF, an agonist that induces Akt activation but weak PKD1 activation in IEC-18 cells (**Fig** **3**, **C**). These results, corroborated by quatification (**Fig** **3**, **D**), demonstrate that the PKD family inhibitor kb NB 142–70 enhances Akt phosphorylation at Thr^308^ and Ser^473^ in response to multiple GPCR agonists in intestinal epithelial IEC-18 cells.

### The selective PKD family inhibitor CRT0066101 and knockdown of PKD1 potentiate GPCR-induced Akt phosphorylation at Thr^308^ and Ser^473^

**Figure 4:**
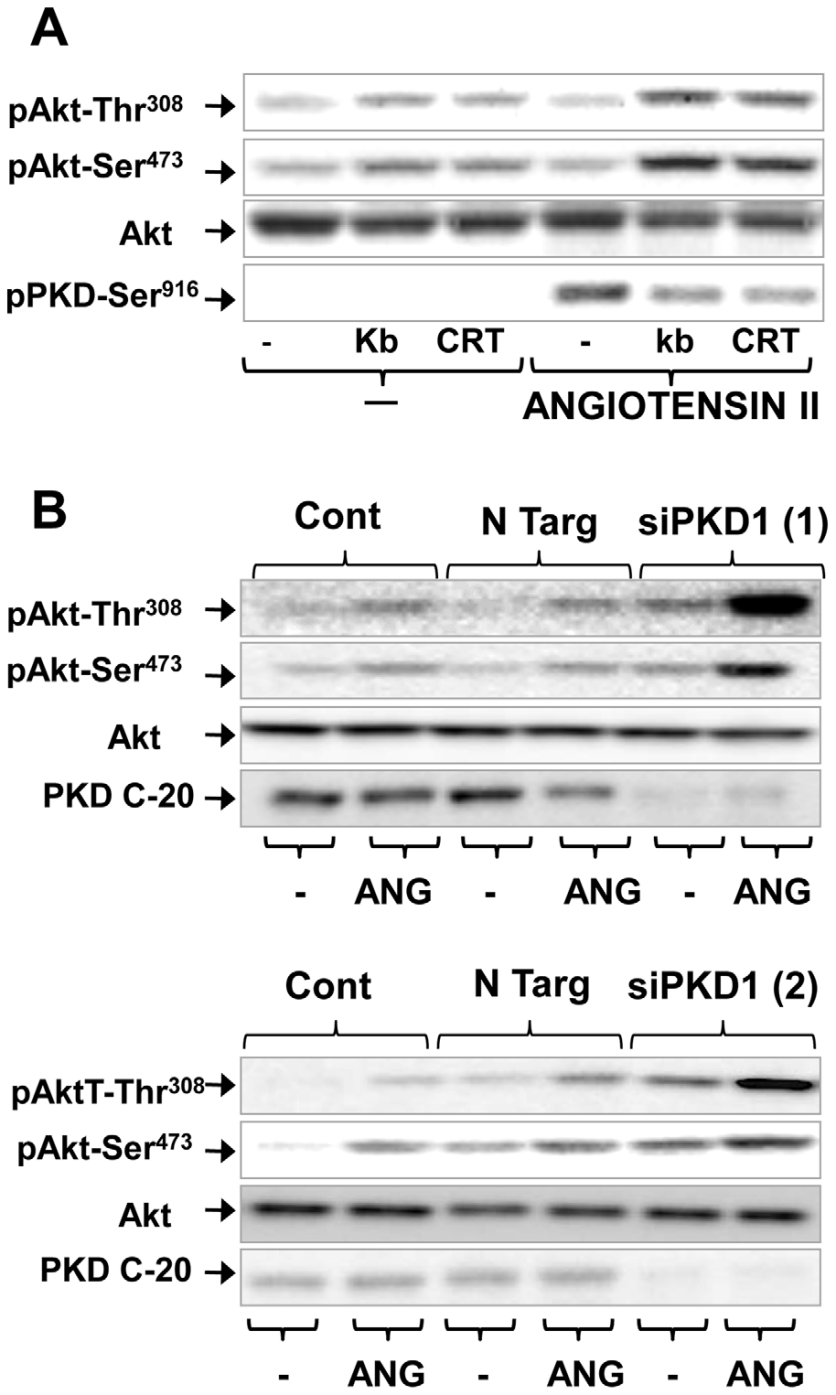
PKD1 mediates feedback inhibition of PI3K/Akt activation in IEC-18 cells stimulated with angiotensin II. **Panel A,** Confluent cultures of IEC-18 cells were incubated in the absence (−) or presence (kb) of either 3.5 µM kb NB 142–70 or 5 μM CRT0066101 (CRT) for 1 h prior to stimulation of the cells with 50 nM angiotensin II (ANGII) for 1 h. All cultures were then lysed with 2× SDS–PAGE sample buffer. The samples were analyzed by SDS-PAGE and immunoblotting with antibodies that detect the phosphorylated state of Akt at Ser^473^ and Thr^308^ and total Akt to verify equal gel loading. PKD1 phosphorylated at Ser^916^ was also determined. **Panels** **B,** Cultures of IEC-18 cells were transfected with non-targeting siRNA (N Targ) or with two different siRNAs targeting PKD1, siPKD1 (1) and siPKD1 (2). Other cultures were not subjected to transfection (Cont). Then, the cultures were stimulated with 50 nM angiotensin II (ANGII) for 10 min and lysed with 2× SDS–PAGE sample buffer. The samples were analyzed by SDS-PAGE and immunoblotting with antibodies that detect the phosphorylated state of Akt at Ser^473^ and Thr^308^, total Akt to verify equal gel loading and total PKD1 (PKD-C20) to evaluate siRNA-mediated knockdown of PKD1 expression. Similar results were obtained in at least 2 independent experiments in each case.

Having established that kb NB 142–70 potentiates Akt activation induced by GPCR agonists, we next determined whether similar enhancing effects can be elicited by a structurally unrelated PKD family inhibitor. We used CRT0066101 [47], a potent and structurally unrelated inhibitor of PKD1 activity in IEC-18 cells [42]. As shown in **Fig.** **4** **A**, treatment with CRT0066101 (5 µM) increased Akt phosphorylation at Thr^308^ and Ser^473^ and inhibited PKD1 activation as potently as kb NB 142–70. These results substantiate the notion that PKD1 inhibition markedly enhances Akt activation in ANG II-stimulated IEC-18 cells.

To prove that PKD1 mediates feedback inhibition of Akt activation in response to GPCR agonits in IEC-18 cells, we determined whether knockdown of PKD1 expression using different siRNAs enhances ANG II-induced Akt phosphorylation at Thr^308^ and Ser^473^ in these cells. Subconfluent cultures of IEC-18 cells were transiently transfected with PKD1 siRNA or nontargeted negative control duplex. To minimize the possibility that the siRNA oligonucleotide may be affecting the expression of a gene other than PKD1, IEC-18 cells were transfected with two different siRNAs targeting distinct regions of PKD1. The PKD1 protein level in IEC-18 cells transfected with either siRNA1 or siRNA2 was dramatically reduced (∼90%) compared with cells transfected with nontargeted negative control duplex (**Fig.** **4** **B**). In contrast, Akt protein levels, determined as a loading control, were not affected. The salient feature of the results shown in **Fig.** **4** **, B** is that PKD1 knockdown by either siRNA1 or siRNA2 strikingly enhanced Akt phosphorylation at Thr^308^ and Ser^473^ in response to ANG II stimulation in IEC-18 cells. Collectively, the results with PKD family inhibitors and siRNAs indicate that PKD1 mediates feedback inhibition of Akt activation in response to GPCR agonists.

### Inhibition of PKD1 increases Akt translocation to the plasma membrane in response to GPCR agonists

**Figure 5:**
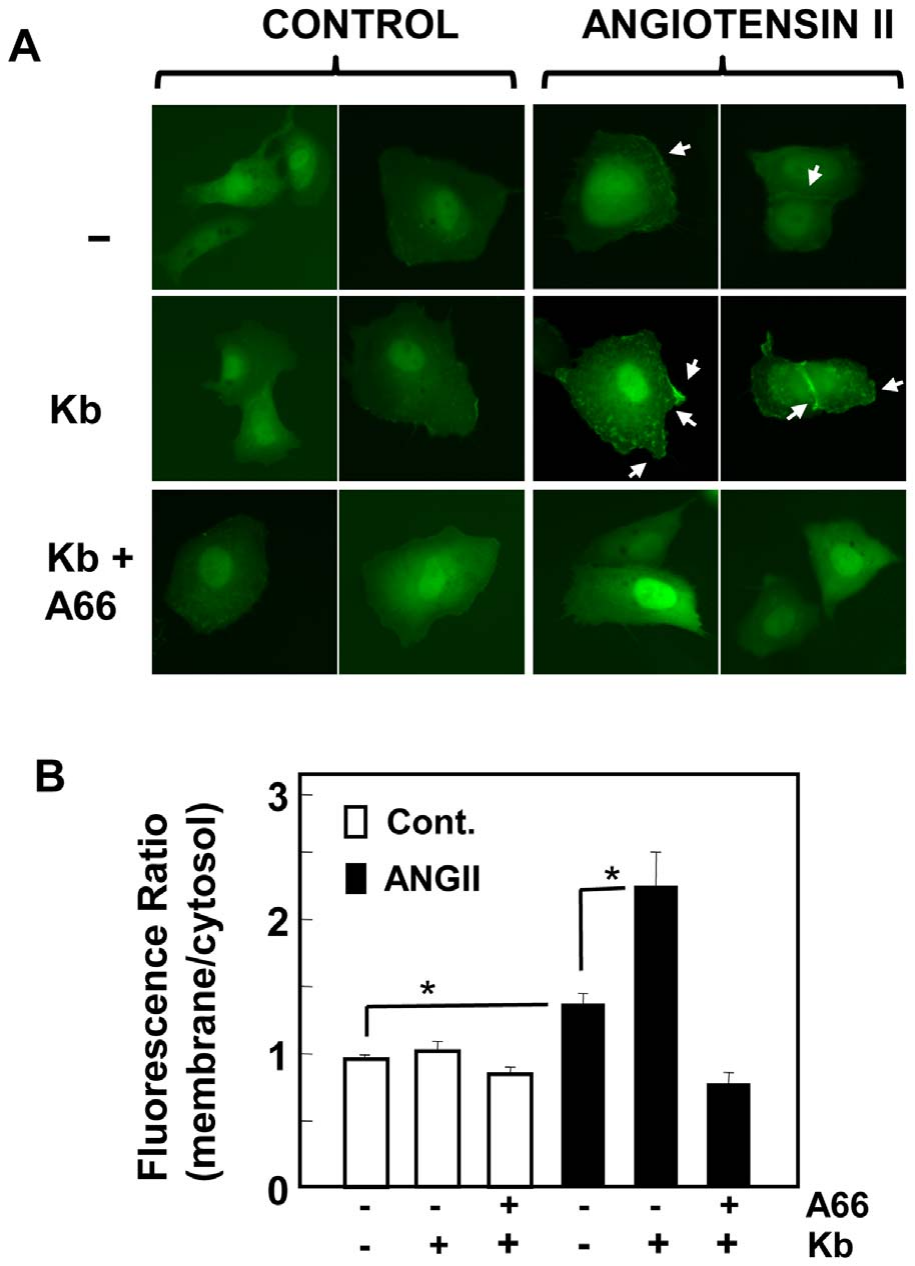
PKD1 inhibition potentiates PI3K-mediated production of PIP_3_ in response to angiotensin II stimulation. IEC-18 cells were transiently transfected with a plasmid encoding a fusion protein between GFP and the PH domain of Akt (Akt-PH-GFP). The cultures were incubated in the in the absence (−) or presence of either 3.5 µM kb NB 142–70 (kb) or 3.5 µM kb NB 142–70 and 10 µM A66 (kb + A66) in DMEM containing 10 mM HEPES for 1 h prior to stimulation with 50 nM angiotensin II (ANG II). The intracellular distribution of Akt-PH-GFP was monitored under a fluorescence microscope, as described in *Materials and Methods*. The selected cells, after 1 h of stimulation with angiotensin II, displayed in the figures were representative of 90% of the population of positive cells. The bars represent the fluorescence ratio (membrane/cytosol) mean ± S.E, n =  at least 20, *p<05.

We next examined the mechanism by which PKD1 restrains Akt activation in response to GPCR agonists in intestinal epithelial cells. Akt phosphorylation at Thr^308^ and Ser^473^ is mediated by protein kinases, PDK1 and mTORC2, that respond to the generation of PIP_3_ by PI3K. The accumulation of PIP_3_ in the plasma membrane initiates Akt translocation to this location, a preriquisite for its subsequent phosphorylation and activation. We considered the possibility that PKD1 activation attenuates production of PIP_3_ and thereby restrains Akt phosphorylation. In order to examine this possibility, we monitored the redistribution of Akt-pleckstrin homology domain-green fluorescent protein (Akt-PH-GFP), an *in vivo* reporter of PIP_3_ [48]. In unstimulated cells, the PIP_3_ sensor was located primarily in the cytosolic compartment without any detectable accumulation at the plasma membrane (**Fig.** **5** **A**). Treatment with ANG II induced detectable translocation of Akt-PH-GFP to the plasma membrane. Prior exposure of the cells to kb NB 142–70 strikingly increased membrane accumulation of the PIP_3_ sensor in response to subsequent stimulation with ANG II (**Fig.** **5** **A**; quatification in **Fig.** **5** **B**). Translocation of Akt-PH-GFP to the plasma membrane was also detected at 5 min and 30 min after ANG II stimulation of IEC-18 cells treated with kb NB 142–70 (**Fig. S2**).

In order to verify that membrane accumulation of Akt-PH-GFP senses PI3K-generated lipid second messengers, we determined whether the recently developed class I p110α specific inhibitor A66 [49] prevents the translocation of Akt-PH-GFP. A66 is a potent inhibitor of p110α but did not affect other class I PI3K isoforms, including p110β, p110δ and p110χ [49].Treatment with A66 completely prevented the translocation of Akt-PH-GFP to the plasma membrane induced by kb NB 142–70 and ANG II (**Fig.** **5** **A**; corroborated by quatification in **Fig.** **5** **B**). These results indicate that exposure to kb NB 142–70 induces a striking increase in PIP_3_ at the plasma membrane via p110α in cells stimulated with ANG II.

### Inhibitors of class I A PI3K and EGFR prevent the potentiation of Akt induced by suppression of PKD1 activity

**Figure 6:**
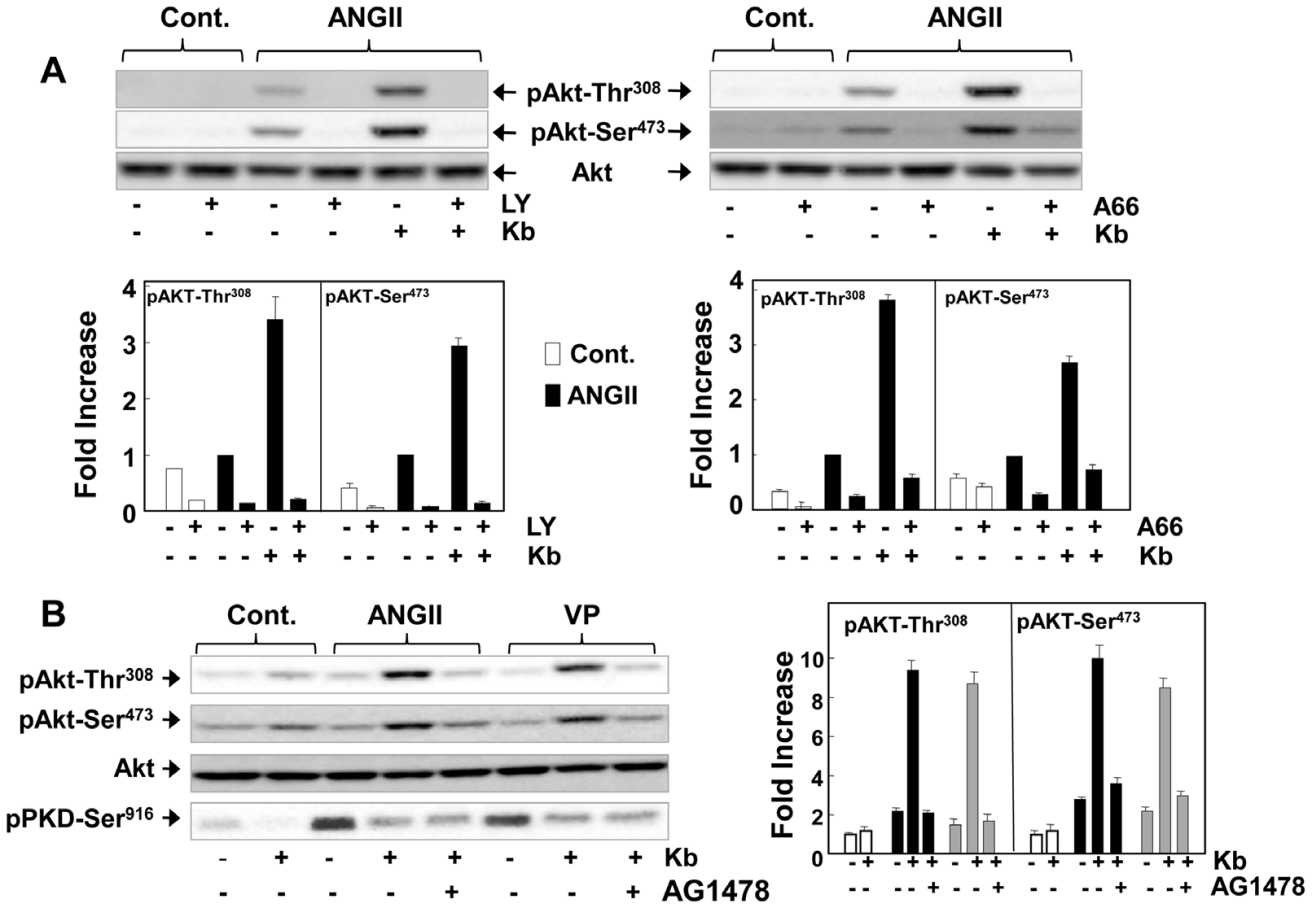
Inhibitors of PI3K and EGFR prevent the potentiation of Akt induced by suppression of PKD1 activity. Confluent cultures of IEC-18 cells were incubated in the in the absence (−) or presence of either 3.5 µM kb NB 142–70 or 3.5 µM kb NB 142–70 and 20 µM LY294002 or 3.5 µM kb NB 142–70 and 10 µM A66 (as indicated) for 1 h prior to stimulation of the cells with 50 nM angiotensin II (ANGII) for 1 h (**Panel A**). Other cultures were incubated in the in the absence (−) or presence of either 3.5 µM kb NB 142–70 or 3.5 µM kb NB 142–70 and 1 µM AG1478 (as indicated) for 1 h prior to stimulation with 50 nM angiotensin II (**Panel B**). All cultures were then lysed with 2× SDS–PAGE sample buffer. The samples were analyzed by SDS-PAGE and immunoblotting with antibodies that detect the phosphorylated state of Akt at Ser^473^ and Thr^308^ and total Akt to verify equal gel loading. PKD1 phosphorylated at Ser^916^ was also determined in panel B. Fold increases in Akt phosphorylations in control (open bars) and in cells stimulated with angiotensin II (black bars) or vasopressin (grey bars) were quantified using Multi Gauge V3.0 and plotted as mean ± S.E.; n = 3.

In view of the preceding results, we next determined whether the increase in Akt phosphorylation by ANGII in cells exposed to kb NB 142–70 is prevented by inhibition of PI3K activity within IEC-18 cells. Treatment with either the PI3K and mTOR inhibitor LY294002 (**Fig.** **6** **A**) or the class IA p110α specific inhibitor A66 (**Fig.** **6** **B**) completely prevented the increase in Akt phosphorylation at Thr^308^ and Ser^473^ in IEC-18 cells exposed to kb NB 142–70 and subsequently challenged with ANG II. Similar results were obtained when the cells were stimulated with vasopressin instead of ANG II (data not shown).

The class IA PI3Ks are heterodimers consisting of a p110 catalytic subunit and a p85 regulatory subunit. Class I A heterodimers involving p110α are activated by tyrosine kinases. The results obtained with the specific p110α inhibitor A66 imply that the striking increase in PIP_3_ accumulation (**Fig.** **5**) and Akt phosphorylation (**Fig.** **6** **, B**) induced by suppression of PKD1 activity in GPCR-stimulated intestinal epithelial cells requires EGFR transactivation. In line with this possibility, treatment of the cells with the specific inhibitor of EGFR tyrosine kinase activity AG1478 completely prevented the enhancement of Akt phosphorylation at Thr^308^ and Ser^473^ in IEC-18 cells exposed to kb NB 142–70 and stimulated with either ANG II or vasoppressin (**Fig.** **6** **C**). These results are consistent with the notion that endogenous GPCRs couple to class IA PI3K involving p110 α via EGFR transactivation in intestinal epithelial IEC-18 cells.

### Role of phosphorylation of the regulatory p85α subunit of PI3K and complex formation of this subunit with EGFR, p110α and PTEN in the negative feedback of PI3K activation mediated by PKD1

**Figure 7:**
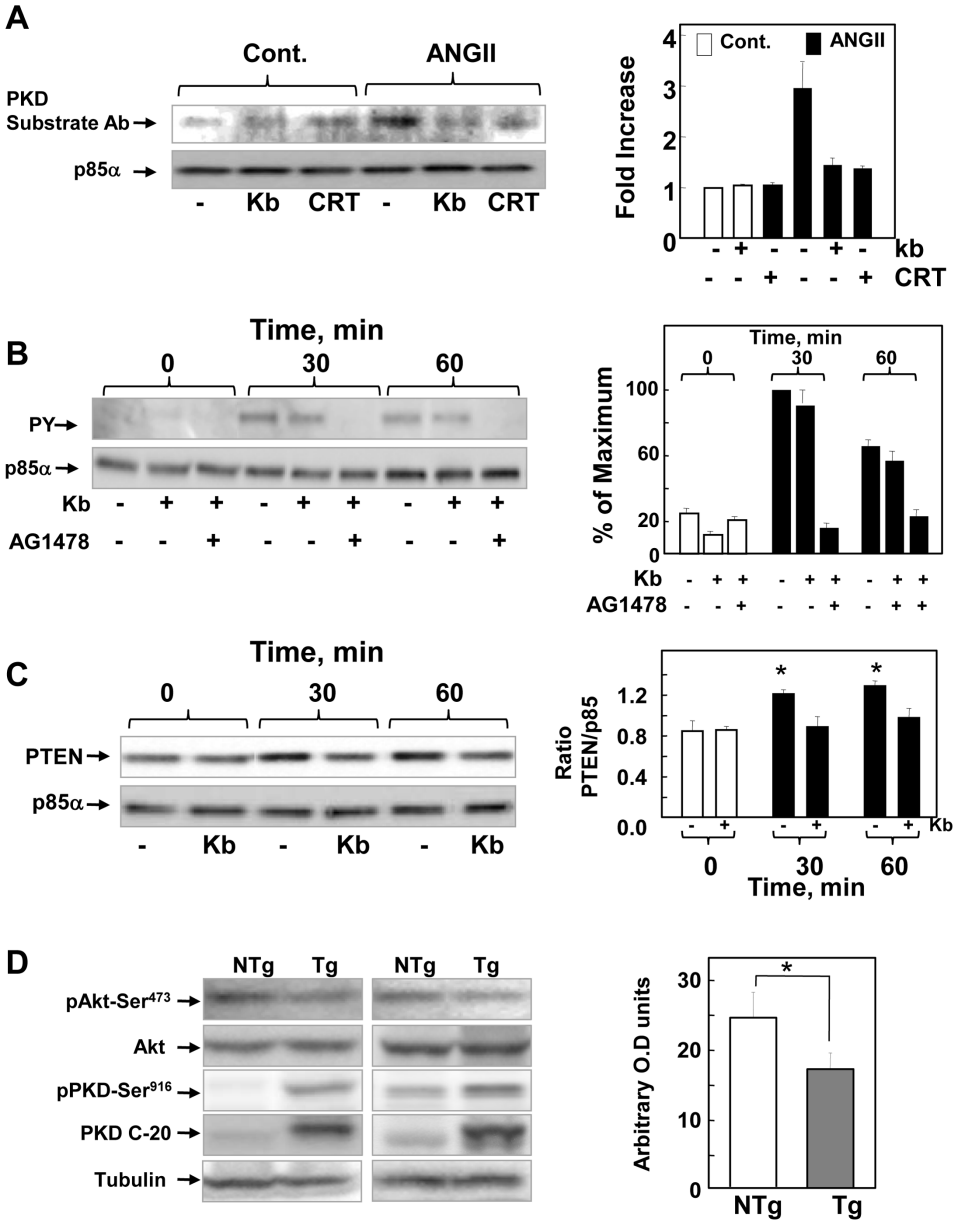
Mechanism of PKD1-mediated negative feedback of PIP_3_/Akt in IEC-18 cells and role of PKD1 in the regulation of Akt phosphorylation *in vivo*. **Panel A, *PKD1-mediated p85α phosphorylation*** Confluent cultures of IEC-18 cells were incubated in the absence (−) or presence (kb) of either 3.5 µM kb NB 142–70 or 5 μM CRT0066101 (CRT) for 1 h prior to stimulation of the cells with 50 nM angiotensin II (ANGII) for 1 h. Cells were lysed and p85α immunoprecipitated as described in Materials and Methods. Immunoblotting was peformed using the PKD substrate motif antibody (Cell Signling Technology). Fold increases in p85α phosphorylation was quantified using Multi Gauge V3.0 and plotted as bars, mean ± S.E; n = 4. **Panel B,** ***p85α complex formation with EGFR*** *.* Confluent cultures of IEC-18 cells were incubated in the absence (−) or presence (kb) of either 3.5 µM kb NB 142–70 or 1 μM AG1478 for 1 h prior to stimulation of the cells with 50 nM angiotensin II (ANGII) for either 30 or 60 min, as indicated. Cells were lysed and p85α immunoprecipitates were analyzed by antiphosphotyrosine immunoblotting. **Panel C,** ***p85α complex formation with PTEN*** Confluent cultures of IEC-18 cells were incubated in the absence (−) or presence (kb) of 3.5 µM kb NB 142–70 for 1 h prior to stimulation of the cells with 50 nM angiotensin II (ANGII) for either 30 or 60 min, as indicated. Cells were lysed and p85α immunoprecipitates were analyzed by immunoblotting with PTEN antibodies. Bars stand for the mean ± S.E n = 4. Individual values are the ratio of PTEN band intensity to the corresponding p85α band intensity in each experiment. *p<0.05 as compared with values at time 0 or values obtained in cells treated with kb NB 142–70 and stimulated with ANG II and at each time point. **Panel D,** ***Overexpression of PKD1 reduces Akt phosphorylation in intestinal epithelial cells*** *.* Epithelial cells from the ileum of transgenic (Tg) mice and nontransgenic (NTg) littermates mice were isolated sequentially by timed incubations in a EDTA-PBS solution. Western blot was used to analyze lysates of these cells for Akt phosphorylated at Ser^473^, total Akt, PKD1 autophosphorylated at Ser^916^ and total PKD1 (PKD-C20). Equivalent loading was verified by immunoblotting for tubulin. Results are shown for 2 transgenic mice and 2 nontransgenic littermates. Bars: represent Akt phosphorylated at Ser^473^ (means ± SE; n = 4). *p<0.05.

Recent results demonstrated that treatment with the phorbol ester PMA inhibited PI3K activation via phosphorylation of the p85α regulatory subunit by PKD1 [43]. Because these studies involved ectopically expressed proteins and phorbol esters are ultrapotent, non-physiological surrogates of endogenous DAG that induce persitent translocation of PKD1 to the plasma membrane, we examined whether physiological PKD1 activation via GPCR-mediated pathways also induces phosphorylation of endogenous p85α. Cultures of IEC-18 cells were treated without or with kb NB 142–70 or CRT0066101, stimulated with ANG II and lysed. The p85α regulatory subunit of PI3K was immunoprecipitated from the lysates and the resulting immunoprecipitates were analyzed by immunoblotting with a motif-specific antibody that detects Ser/Thr phosphorylated by PKD family members (i.e. Ser/Thr phosphorylated within a LXRXX Ser/Thr sequence). In silico analysis and recent experimental results [43], [44] indicate that the PI3K regulatory subunit p85α contains several consensus PKD phosphorylation motifs, including Ser^154^, Ser^361^ and Ser^652^ As shown in **Fig. 7** **A**, stimulation of cells with ANG II markedly increased the phosphorylation of p85α detected by the PKD motif-specific antibody. We verified that similar amounts of p85α were immunoprecipitated from lysates of cells exposed to different treatments. Crucially, the increase in the phosphorylation of p85α induced by stimulation with ANG II was completely prevented by prior cell exposure to the PKD family inhibitors kb NB 142–70 and CRT0066101 (**Fig. 7** **A**). The results indicate that GPCR activation induces phosphorylation of the endogenous p85α regulatory subunit of PI3K through PKD1 in intestinal epithelial cells.

To explore the mechanisms by which PKD1-mediated phosphorylation of the p85α subunit attenuates PIP_3_ accumulation and Akt phosphorylation, we examined possible molecular events, including (1) inhibition of the binding of p85α to tyrosine phosphorylated residues; (2) complex formation between p85α and either p110α or PTEN. In order to determine whether PKD1-mediated phosphorylation of p85α inhibits its binding to the EGFR, cultures of IEC-18 cells treated without or with kb NB 142–70 or the specific EGFR tyrosine kinase inhibitor AG1478. Subsequently, the cultures were stimulated with ANG II for various times, lysed and p85α immunoprecipitates were analyzed by anti-phosphotyrosine immunoblotting. As shown in **Fig. 7** **B**, stimulation with ANGII induced rapid binding of p85α to a major tyrosine phosphorylated band migrating with the apparent molecular mass of the EGFR (185,000 kDa). The detection of this band was completely extinguished by treatment with AG1478, indicating that this band corresponds to the tyrosine phosphorylated EGFR. These results corroborated that GPCR stimulation of IEC-18 cells induces transactivation and tyrosine phosphorylation of the EGFR leading to complex formation with the p85α subunit of PI3K. Our results also show that binding of p85α to the EGFR was not altered by treatment with kb NB 142–70, implying that the increase in PI3K activity in response to PKD1 inhibition is not mediated by enhanced EGFR/p85α complex formation. Anti p110a immunoblotting of p85a immunoprecipitates showed that treatment with kb NB 142–70 did not alter the level of the p85α/p110α complex in quiescent and stimulated cells (**Fig. S3**).

A recent study demonstrated that PKD1 phosphorylates p85α at Ser^154^ to enhance its interaction with PTEN, thereby leading to PTEN activation in neutrophiles [44]. Here, we examined whether PKD1 stimulates binding of p85α to PTEN in intestinal epithelial cells. We found that stimulation of IEC-18 cells with ANG II enhanced p85α/ PTEN complex formation in IEC-18 cells, as determined by PTEN immunoblotting of p85α immunoprecipitates (**Fig. 7** **C**). A salient feature of these results is that prior exposure of the cells to kb NB 142–70 prevented the increase in p85α/PTEN complex formation induced by ANG II (**Fig. 7** **C**). The results suggest that PKD1-mediated phosphorylation of p85α mediates negative feedback of PIP_3_ accumulation and Akt phosphorylation in GPCR-stimulated cells, at least in part, by enhancing the stimulatory association of p85α with PTEN.

### Overexpression of PKD1attenuates Akt phosphorylation at Ser^473^ in intestinal epithelial cells *in vivo*

Collectively, our preceding results indicating that PKD1 mediates negative feedback of PI3K/Akt activation in IEC-18 cells prompted us to hypothesize that PKD1 regulates PIP_3_ levels and Akt activity of intestinal epithelial cells *in vivo*. To test this hypothesis, we used transgenic mice that express elevated PKD1 protein in the small intestine epithelium [37]. We verified overexpression of PKD1 protein in the ileum of PKD1 Tg mice by Western blot analysis of total PKD1 in lysates of epithelial cells (**Fig. 7** **D**). Overexpressed PKD1 was active, as revealed by Ser^916^ autophosphorylation. The salient feature of the results is that overexpression of PKD1 was associated with reduced phosphorylation of Akt at Ser^473^ in intestinal epithelial cells *in vivo* (**Fig. 7** **D**). Collectively, our results indicate that PKD1 mediates negative feedback of PI3K/Akt activation within intestinal epithelial cells *in vitro* and *in vivo*.

## Discussion

GPCR agonists act as potent cellular growth factors and have been implicated in a variety of normal and abnormal processes, including development, inflammation, and malignant transformation [50], [51]. Despite the fundamental importance of GPCR-mediated biological responses in normal and abnormal cell regulation, the regulatory mechanisms involved remain incompletely understood. Here, we examined the hypothesis that PKD1 mediates negative feedback that regulates the intensity and duration of PI3K/Akt signaling. Using intestinal epithelial IEC-18 cells as a model system of GPCR-induced transient Akt activation [6], we produced here several lines of evidence indicating that PKD1 mediates feedback inhibition of PI3K/Akt activation. Specifically: **1**) Treatment with the preferential PKD family inhibitor kb NB 142–70, at concentrations that inhibited PKD1 activation, induced a striking enhancement of Akt phosphorylation at Thr^308^ and Ser^473^ in response to the Gq-coupled receptor agonist ANG II; **2**) Enhancement of Akt activation was elicited in cells challenged with various concentrations of ANG II or with a fixed concentration of ANG II for various times. These studies revealed that inhibition of PKD family activity converted Akt signaling from transient to persistent; **3**) Exposure to kb NB 142–70 enhanced Akt phosphorylation in IEC-18 cells stimulated with the GPCR agonists vasopressin or LPA as potently as in paralell cultures stimulated with ANG II. In contrast, kb NB 142–70 had only a small potentiating effect on Akt activation induced by EGF, an agonist that induces weak PKD1 activation in IEC-18 cells; **4**) We verified that the potentiating effect of kb NB 142–70 on Akt phosphorylation can be mimicked by cell treatment with a structurally unrelated inhibitor of the PKD family, namely CRT0066101; **5**) Extensive knockdown of PKD1 expression using siRNAs directed against different regions of PKD1 enhanced ANG II-induced Akt phosphorylation at Thr^308^ and Ser^473^. These results demonstrate, for the first time, that endogenous PKD1 mediates potent feedback inhibition of GPCR-induced Akt activation.

Since PKD1 mediates negative feedback of PI3K/Akt activation in IEC-18 cells, we hypothesized that PKD1 regulates Akt activity of intestinal epithelial cells *in vivo*. To test this hypothesis, we used transgenic mice that express elevated PKD1 protein in the small intestine epithelium [37]. We found that overexpression of PKD1 was associated with reduced phosphorylation of Akt at Ser^473^ in intestinal epithelial cells. Collectively, the results indicate that PKD1 mediates negative feedback of Akt activation within intestinal epithelial cells *in vitro* and *in vivo*.

In order to examine the mechanism by which PKD1 attenuates Akt activation, we evaluated the activity of PI3K in single cells by monitoring the redistribution of Akt-PH-GFP, an *in vivo* reporter of PIP_3_ [48]. We found, for the first time that exposure of intestinal epithelial cells to the PKD family inhibitor kb NB 142–70 strikingly increased the translocation of Akt-PH-GFP from the cytosol to the plasma membrane in ANG II-stimulated cells. The redistribution of the PIP_3_ sensor and the enhancement of Akt phosphorylation at Thr^308^ and Ser^473^ were completely blocked by exposure to the class I p110α specific inhibitor A66. Class IA PI3K heterodimers involving p110α are activated by tyrosine kinases, implying that PIP_3_ accumulation and Akt activation induced by suppression of PKD1 activity in GPCR-stimulated intestinal epithelial cells require GPCR-induced EGFR transactivation. Accordingly, treatment with the specific inhibitor of EGFR tyrosine kinase activity AG1478 completely prevented the enhancement of Akt phosphorylation in cells exposed to kb NB 142–70 and stimulated with either ANG II or vasoppressin. Consequently, we conclude that PKD1 mediates negative feedback of GPCR-induced Akt activation by preventing PIP_3_ accumulation in the plasma membrane, the second messenger that triggers the translocation and subsequent phosphorylation of Akt.

As mentioned before, the class IA PI3Ks are heterodimers consisting of a p110 catalytic subunit and a p85 regulatory subunit [52]. The binding of the SH2 domains of the p85 subunit to tyrosine phosphorylated residues in receptors (e.g. transactivated EGFR) alleviates the p85-mediated inhibition of p110 isoforms and also brings them in contact with their lipid substrates in the membrane [2]. A number of studies demonstrated that p85α is phosphorylated on serine residues by autophosphorylation at Ser^608^ [53], [54] and by transphosphorylation on other sites by other protein kinases [43], [55], [56]. Recent results demonstrated that treatment with the phorbol ester PMA inhibited PI3K activation via phosphorylation of the p85α regulatory subunit by PKD1, predominantly at Ser^652^ [43]. The phosphorylation of residues Ser^361^ and Ser^652^ interfered with PI3K activation. However, these studies used ectopically expressed proteins and phorbol esters, which are ultrapotent, non-physiological surrogates of rapidly turning over endogenous DAG. Furthermore, phorbol esters induce PKD1 activation via PKC at early and late times [36], rather than through rapid PKC-dependent phase followed by a PKC-independent phase, as shown in cells stimulated via GPCRs [36]–[38] or in response to Gq activation [30]. Consequently, the significance of the results with phorbol esters remained unclear. A recent study demonstrated that PKD1 phosphorylates p85α at Ser^154^ to enhance its interaction with PTEN, thereby regulating neutrophil migration [44]. However, the impact of PKD1 activity on the cellular levels of PIP_3_ has not been examined in any of the previous studies.

To asses the importance of p85α phosphorylation in the mechanism by which PKD1 attenuates PIP_3_ accumulation and Akt phosphorylation in response to GPCR activation in intestinal epithelial cells, we determined whether physiological PKD1 activation via GPCR-mediated pathways also induces phosphorylation of endogenous p85α. Here, we demonstrate that stimulation of IEC-18 cells with ANG II markedly increased PKD1-mediated phosphorylation of p85α in response to ANG II, as shown with a PKD family motif-specific antibody. The immureactive band detected by this antibody was completely extinguished by cell exposure to the PKD family inhibitors kb NB 142–70 or CRT0066101. Our results support a model in which PKD1 inhibits PIP_3_ accumulation within intestinal epithelial cells stimulated with GPCR agonists by phosphorylating the p85α regulatory subunit of class IA PI3Ks. We examined several potential mechanisms by which PKD1-mediated phosphorylation of p85α attenuates PIP_3_ accumulation. Co-immunoprecipitation experiments indicated that suppresion of PKD1 activity did not enhance EGFR/p85α complex formation or change the level of the p85α/p110α PI3K heterodimer. In contrast, we found that GPCR activation induced p85α binding to PTEN and inhibition of PKD1 prevented p85α/PTEN complex formation. Consequently, we conclude that PKD1-mediated phosphorylation of p85α mediates negative feedback of PIP_3_ accumulation and Akt phosphorylation, at least in part, by enhancing the stimulatory association of phosphorylated p85α with PTEN leading to PIP_3_ dephosphorylation.

The regulation of the intensity and duration of signaling pathways is of critical importance for determining cellular outcomes, including metabolism, proliferation, survival, growth arrest or senescence. While this notion is well supported by studies of the RAF/MEK/ERK pathway [35], [51], [57], [58], increasing evidence also points to a critical role of the duration of PI3K/Akt signaling in cellular regulation. Constitutive activation of Akt promotes senescence in a variety of cell types [16], including endothelial progenitors, mouse embryonic fibroblasts [59]–[61] and mouse prostate epithelial cell [60] and links diet-induced obesity with vascular senescence and cardiovascular disease [62]. Constitutive activation of Akt promotes senescence-like arrest of cell growth via a p53/p21-dependent pathway, and inhibition of forkhead transcription factor FOXO3a by Akt is essential for this growth arrest to occur [19]. Furthermore, the intensity of Akt activation modulates NF-kB-mediated gene expression [63]. Conversely, sustained inhibition of PI3K/Akt induces expression and activation of multiple tyrosine kinase receptors [20], [21]. These findings indicate that the duration and intensity of the PI3K/Akt signaling play a critical role in determining cellular outcomes. These observations, obtained in a variety of model systems, imply that a fine balance of Akt activity is of critical importance in cell regulation and emphasize the importance of feedback loops that contribute to adjust PIP_3_ levels within cells. Based on the results presented here, we propose that PKD1 mediates negative feedback of PIP_3_ accumulation thereby contributing to dynamic PIP_3_/Akt signaling in the cell. Together with previous results demonstrating that PKD1 prolongs ERK activation but attenuates JNK [23], PKD1 emerges as a critical node in the control of the intensity and duration of signal transduction pathways of fundamental importance in cell regulation.

## Supporting Information

Figure S1 (PDF)

Figure S2 (PDF)

Figure S3 (PDF)
