# Correction: PKD1 Mediates Negative Feedback of PI3K/Akt Activation in Response to G Protein-Coupled Receptors

**DOI:** 10.1371/annotation/70d3c7b0-9718-4bb1-abed-d0defc3b7fc4

**Published:** 2014-01-15

**Authors:** Yang Ni, James Sinnett-Smith, Steven H. Young, Enrique Rozengurt

During production, an error was introduced to the fourth sentence of the Abstract. The correct sentence reads: 

"Cell treatment with the structurally unrelated PKD family inhibitor CRT0066101 increased Akt phosphorylation as potently as kb NB 142-70."

