# Supplementary figures and images for: PKD1 Mediates Negative Feedback of PI3K/Akt Activation in Response to G Protein-Coupled Receptors
Source: PLoS One. 2013 Sep 9;8(9):e73149. doi: 10.1371/journal.pone.0073149 (PMC3767810; doi:10.1371/journal.pone.0073149)

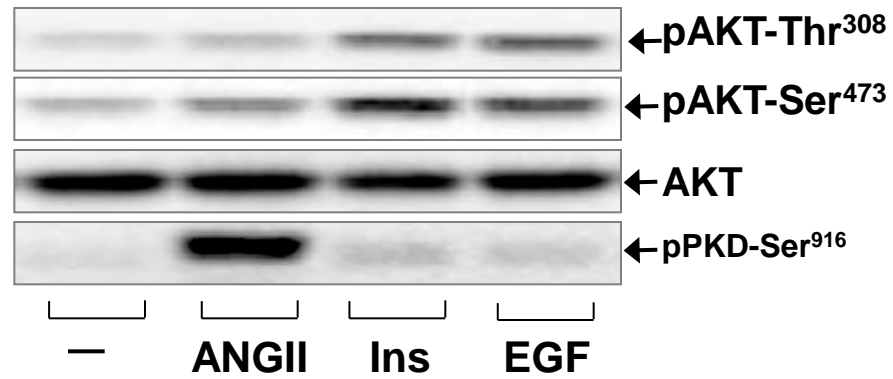

Supplement: Figure S1 — (PDF) [file pone.0073149.s001.pdf]

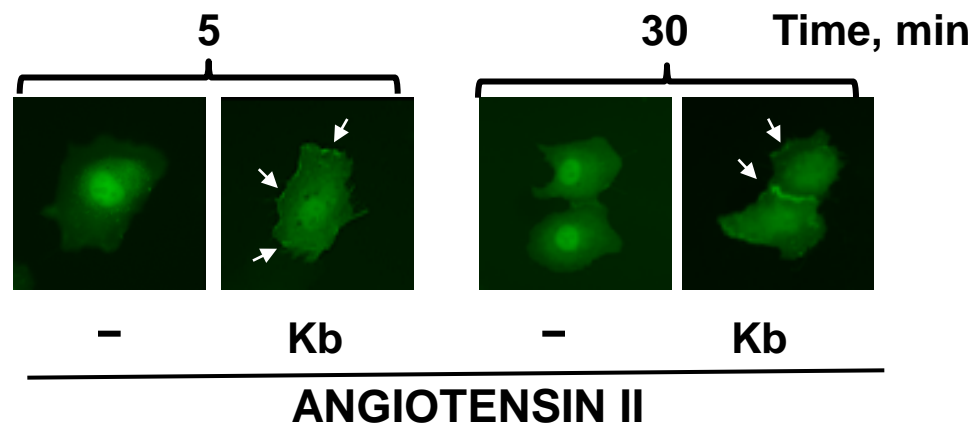

Supplement: Figure S2 — (PDF) [file pone.0073149.s002.pdf]

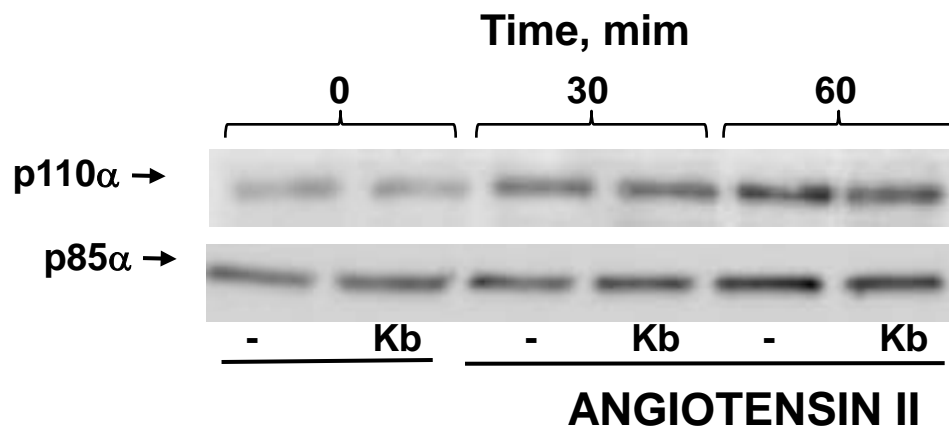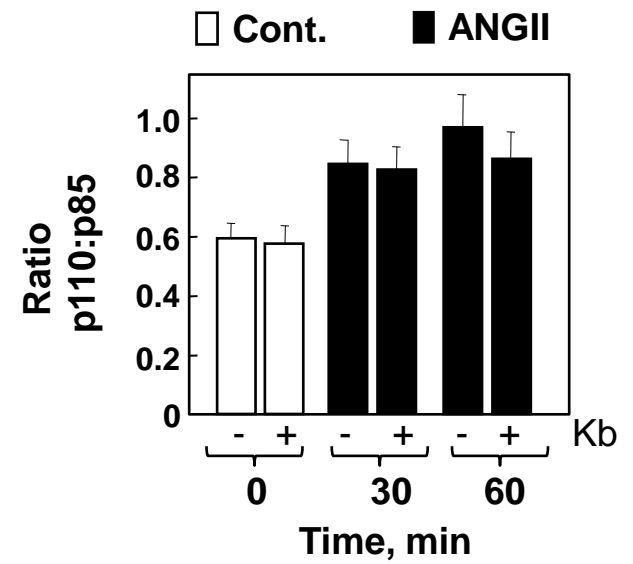

Supplement: Figure S3 — (PDF) [file pone.0073149.s003.pdf]
